# Three-dimensional longitudinal assessment of nasal development with and without Nasoalveolar molding therapy in cleft lip and palate patients

**DOI:** 10.1007/s00784-025-06589-7

**Published:** 2025-10-03

**Authors:** Lucas M. Ritschl, Klaudia Narbekovas, Denys J. Loeffelbein, Alex Grabenhorst, Nils Krautkremer, Hannes Singer, Andrea Grandoch, Helena Kram, Klaus-Dietrich Wolff, Florian D. Grill

**Affiliations:** 1 Department of Oral and Maxillofacial Surgery, School of Medicine and Health, Technische Universität München, Munich, Germany; 2 Department of General Surgery, Medical University of Vienna, Vienna, Austria; 3 Department of Oral and Maxillofacial Plastic Surgery, Helios Klinikum München West, Academic Teaching Hospital of Ludwig-Maximilians- Universität München, Munich, Germany; 4 Department of Oral and Maxillofacial Surgery, University Clinic of Cologne, Cologne, Germany; 5 Praxisklinik für Mund-, Kiefer-, und Plastische Gesichtschirurgie, Wolfratshausen, Germany

**Keywords:** Cleft lip and palate, Presurgical nasoalveolar molding, Conventional palatal plate treatment, Nasal symmetry

## Abstract

**Background:**

Grayson-type presurgical nasoalveolar molding (PNAM) is an established method in presurgical infant orthopedics. This study compared PNAM therapy with conventional palatal plates across cleft types, focusing on nasal morphology.

**Methods:**

Seventy-two patients with non-syndromic unilateral (UCLP) and bilateral cleft lip and palate (BCLP) were followed from birth to two years. Three-dimensional (3D) models of the perinasal area were obtained via cast-based scans or 3D photographs; right-sided UCLP cases were mirrored to the left. Two independent reviewers analyzed anatomical landmarks with excellent inter-rater reliability (ICC = 0.88). Initial and two-year measurements were compared between PNAM (NAM group) and conventional treatment (non-NAM group).

**Results:**

Of the 72 patients, 46 had UCLP and 26 BCLP. In BCLP, NAM therapy promoted greater columella elongation from 2.1 mm at T_0_ to 5.2 mm at T_3_ compared to a change from 2.4 mm to 3.5 mm in the non-NAM group (*p* = 0.300). In UCLP, NAM was also associated with increases in columella (*p* = 0.387) and improved columella deviation angles, from a median of 48.1° at T_0_ to 85.6° at T_3_, while non-NAM showed a modest increase from 70.6° to 79.1°, (*p* = 0.167) Significant reductions were observed cleft-side nostril length (*p* = 0.027) and width (*p* = 0.029).

**Conclusion:**

This study, using 3D imaging, demonstrates the clinically relevant capability of PNAM to improve nasal morphology compared to conventional treatment, potentially enhancing primary cheiloplasty outcomes. The longitudinal design three-dimensionally tracks preoperative and postoperative changes over two years, with further investigation needed for long-term stability.

## Introduction

Cleft lip and palate (CLP) are among the most prevalent congenital anomalies, affecting approximately one out of 700 births globally [1, 2]. They can present unilaterally or bilaterally and are associated with challenges in feeding, speech development, and facial appearance, requiring multidisciplinary care [3].

Early presurgical interventions play an important role in improving functional and esthetic outcomes. Conventional palatal plates have long been used to facilitate feeding and support maxillary growth. In the 1990 s, PNAM was introduced as a presurgical supportive treatment with the additional aims of molding the nasal cartilages and alveolar ridges [4, 5]. A prospective 3D analysis by Chou et al. demonstrated significant progressive improvements in nasal symmetry and columella length in infants with complete UCLP following NAM therapy [6–10]. However, much of the available evidence is based on short-term follow-up and focuses primarily on maxillary arch changes rather than nasal morphology.

Traditionally, facial documentation has relied on two-dimensional photography and plaster models, which are limited by issues of accuracy and reproducibility [11–14]. The introduction of three-dimensional (3D) imaging methods has greatly improved the ability to document and assess the oral and maxillofacial morphology of CLP patients compared to traditional techniques such as direct anthropometry and two-dimensional photography [15–18]. Mancini et al. conducted a retrospective longitudinal study using 3D stereophotogrammetry in infants with BCLP, documenting significant improvements in nasolabial symmetry and columella length after NAM and primary lip/nose surgery [19]. However, too few studies have used objective 3D imaging techniques with longer observation periods to assess their comparative effects against conventional plates, particularly across UCLP and BCLP groups [20, 21]. This study tests the null hypothesis that NAM therapy has no significant effect on nasal morphology. The aim is to assess nasal development across different cleft types by comparing outcomes between NAM and non-NAM treatment groups, using both 3D photographic imaging and 3D digital models to evaluate nasal shape and symmetry. The results show the effectiveness of NAM therapy in improving nasal morphology and symmetry, greater increases in columella length, and nasal tip protrusion in infants with UCLP and BCLP.

Therefore, the aim of this study was to evaluate the effect of NAM therapy on nasal morphology in infants with UCLP and BCLP compared to conventional palatal plate treatment, using 3D model analysis. The null hypothesis tested was that NAM therapy has no significant impact on nasal development compared to conventional palatal plate treatment.

## Materials and methods

### Study design

This single-center longitudinal observational study was partly performed on a historical cohort. For this purpose, plaster models starting from the year 2014 were digitized. Further, between the years 2019 and 2023 patients were actively enrolled for impression-taking and 3D photographs. The plaster models were then also digitized.

The study combined retrospective plaster models with prospectively collected 3D scans. As plaster impressions are prone to soft-tissue distortion and digitization error, while direct 3D imaging provides higher consistency, this methodological heterogeneity may have introduced differences in data quality and is acknowledged as a potential source of bias [22].

### Ethical statement

The study was conducted in accordance with the 1964 Declaration of Helsinki. Ethical approval was obtained from the Ethics Committee of the Technical University of Munich (approval number: 275/18 s). Written informed consent was obtained from the parents or legal guardians of all participating infants.

### Participants

Only patients with non-syndromic UCLP and non-syndromic BCLP were included in the study, based on the following inclusion and exclusion criteria: treatment conducted exclusively at the Department of Oral and Maxillofacial Surgery at the School of Medicine, Technical University of Munich, Klinikum rechts der Isar; diagnosis of a complete unilateral or bilateral cleft lip and palate; completion of presurgical plate therapy (either NAM or non-NAM); provision of informed consent.

Exclusion criteria were incomplete NAM therapy, treatment outside the up-mentioned institution, no informed consent, or the presence of syndromic conditions.

Treatment allocation was based on parental choice after standardized counseling. This approach reflects real-world clinical practice but introduces potential selection bias, as socio-economic status, parental motivation, or treatment compliance may have influenced the decision between NAM and conventional plate therapy.

### Intervention NAM group

**Fig. 1:**
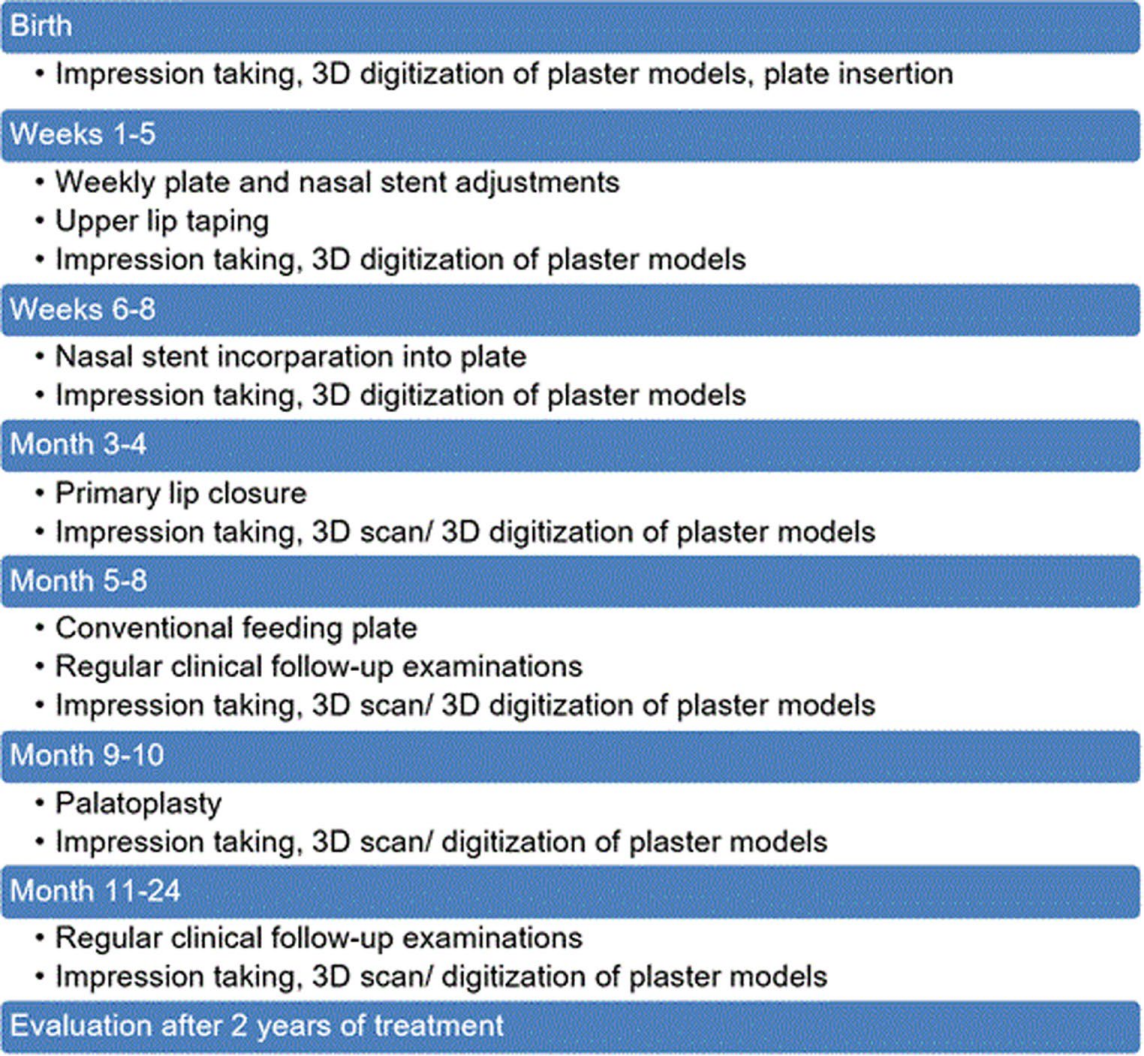
Schematic Workflow of the Nasoalveolar Molding Procedure

Treatment was assessed as follows: An impression was taken within the first days after birth to produce a feeding plate, and parents were informed about the cleft lip and palate treatment protocol. If NAM therapy was chosen by the parents, it began within the first week using Grayson’s technique, with weekly check-ups as reported earlier [23, 24]. The plate was adjusted to approximate the alveolar ridges and support jaw development, aided by upper lip taping. After 6–8 weeks, the plate is further expanded with a nasal bridge. NAM therapy ended with primary lip closure at 3–4 months for which the anesthesiologists in our institution require a body weight of at least 5 kg. Postoperatively, conventional feeding plates were used until palatal closure. The workflow is shown in Fig. 1.

### Intervention non-NAM group

If the parents chose a treatment with conventional intraoral molding plates, the therapy starts with impression-taking of the jaw and insertion of a palatal plate. The plate is made individually for each patient and should ideally be worn by the baby all day and at night, and always when feeding.

### Study population

The final study population consisted of 72 patients with cleft lip and palate. Of these, 26 patients were diagnosed with BCLP, of whom 19 received NAM therapy. Forty-six patients presented with UCLP, with 36 patients having received NAM therapy. An overview of the patient group distributions is provided in Table 1. For consistent comparison, all right-sided UCLPs were digitally mirrored to the left side using GOM ^®^ Inspect 3D software (Version 2021, GOM ^®^ GmbH, Braunschweig, Germany).

**Table 1:** Patient distribution by cleft type and therapy group (BCLP = bilateral cleft lip and palate, UCLP = unilateral cleft lip and palate)

Cleft Type	Number of Patients	NAM Therapy	Non-NAM Therapy
BCLP	26	19	7
UCLP Right	13	11	2
UCLP Left	33	25	8
Total UCLP	46	36	10
Total (UCLP + BCLP)	72	55	17

### Documentation

All data were generated in the Department of Oral and Maxillofacial Surgery at the School of Medicine, Technical University of Munich, Klinikum rechts der Isar. For both abovementioned treatment groups, impressions and 3D scans were taken at these four different time points: T0 (0–50 days), T1 (51–100 days), T2 (101–250 days), and T3 (251–650 days).

### Plaster models

Impressions were taken with a semitransparent A-silicone (Memosil ^®^ 2; Kulzer GmbH, Germany) 25 by experienced clinicians in a controlled clinical setting [25]. Infants were positioned in a semi-upright posture, supported either by a caregiver or trained clinical staff to maintain airway patency and minimize movement. There was a continuous visual monitoring to immediately detect respiratory difficulties.

After impression removal, the oral cavity was inspected to exclude residual material and prevent aspiration. Plaster models were digitized with a 3D triangulation scanner (3Shape D500 Dental System™ ^®^ Standard, 3Shape A/S, Horsens, Denmark, Software Dental System™ 2014 – Premium) and saved as.stl files.

### 3D imaging

Additionally, 3D imaging was used to quantify changes in nasal growth in cleft patients. All scans were taken with the Artec ^®^ Space Spider (Artec ^®^ Group; Luxembourg). 3D imaging was used to assess nasal growth in cleft patients, with scans taken using the Artec^®^ Space Spider (Artec Group, Luxembourg). Infants were positioned semi-upright and stabilized to minimize motion. Scans were performed at 40–50 cm under consistent lighting, with the scanner calibrated before each session. Images were captured in a calm setting with a neutral facial expression.

### Landmark analysis

**Fig. 2:**
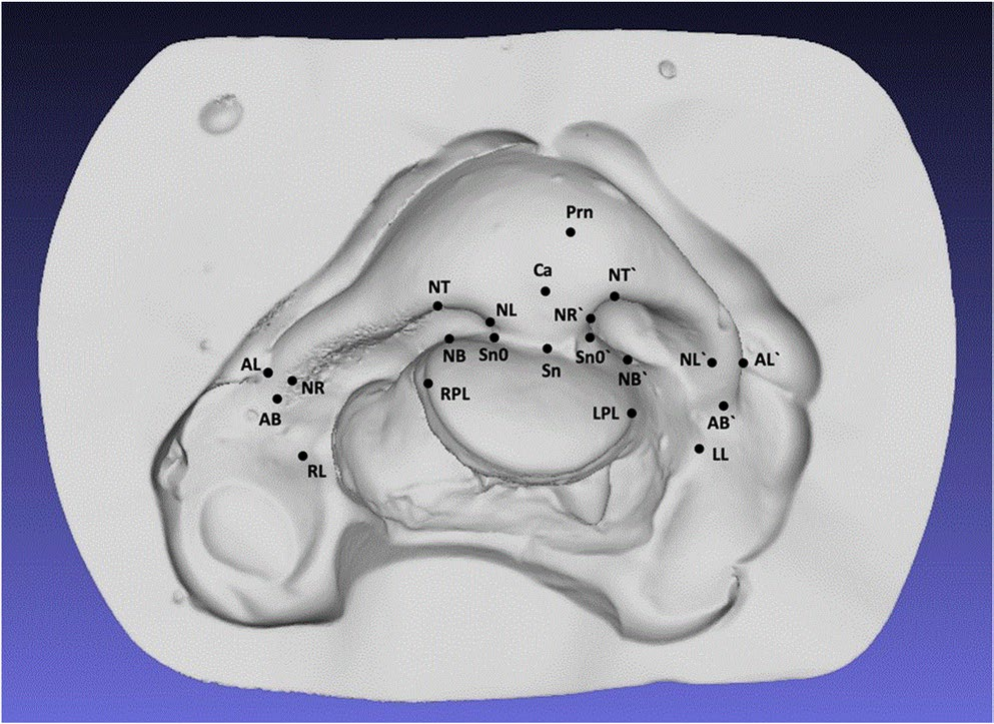
Location of all 21 Landmarks Used Exemplarily on a Digitized Plaster Model of a BCLP Patient

**Fig. 3:**
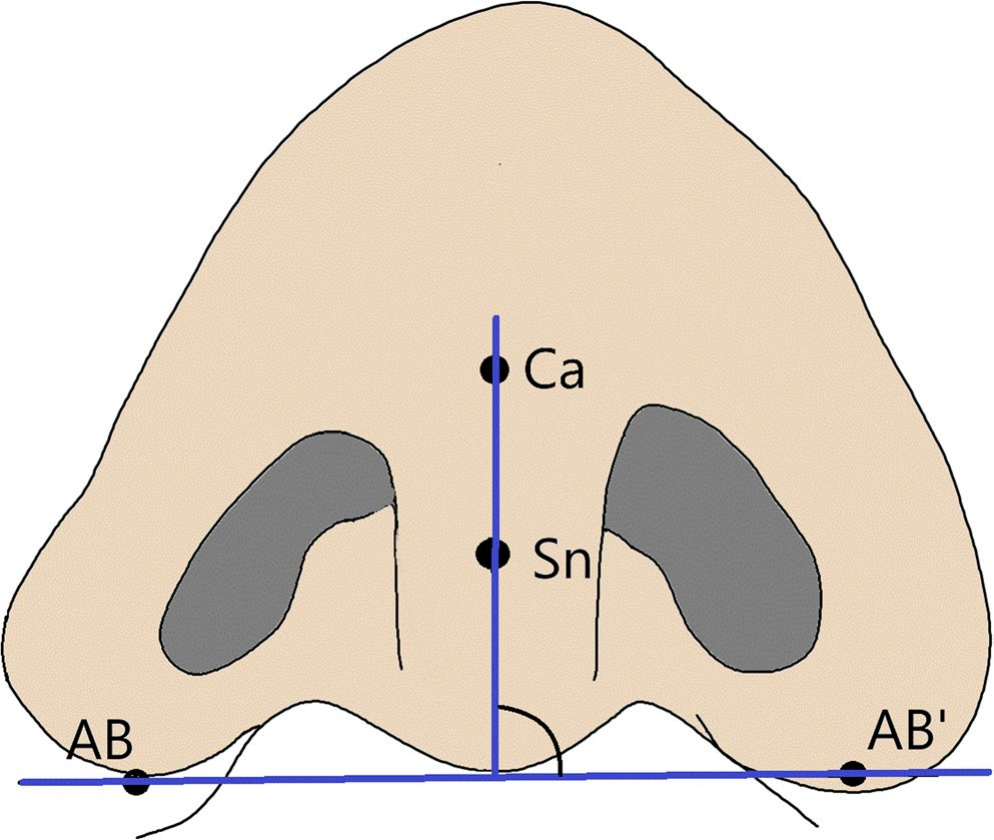
Nose Bottom View Showing Landmarks for the Nasal Baseline and Columella Length

Mirrored models of right-sided CLP were created with GOM^®^ Inspect (Version 2021, GOM GmbH, Braunschweig, Germany) to standardize all unilateral cases to the left side. Digitized plaster models were analyzed virtually using predefined landmarks from prior literature [26, 27]. A reference plane, defined by the columella and alar bases, was used to assess columella deviation and nasal symmetry. Landmarks, 3D measurements, and abbreviations are shown in Figs. 2 and 3; Table 2.

**Table 2:** Definitions and abbreviations of measured 3D distances and angles

3D Distance	Abbreviation	Definition
Nose width	AL – AL′	Distance from right alare (AL) to left alare (AL′)
Protrusion of nasal tip	Sn – Prn	Distance from subnasale (Sn) to pronasale (Prn)
Columella length	Sn – Ca	Distance from subnasale (Sn) to anterior columella point (Ca)
Columella thickness	Sn₀ – Sn₀′	Distance from right subnasale (Sn₀) to left subnasale (Sn₀′)
Nostril height (right)	NB – NT	Distance from lowest (NB) to highest (NT) point of the right nostril
Nostril height (left)	NB′ – NT′	Distance from lowest (NB′) to highest (NT′) point of the left nostril
Nostril width (right)	NR – NL	Distance from farthest right (NR) to farthest left (NL) point of right nostril
Nostril width (left)	NR′ – NL′	Distance from farthest right (NR′) to farthest left (NL′) of left nostril
Nostril length (right)	AB – Prn	Distance from alar base (AB) to pronasale (Prn) – right side
Nostril length (left)	AB′ – Prn	Distance from alar base (AB′) to pronasale (Prn) – left side
Width of cleft lip (right)	RL – RLP	Distance from right cleft lip (RL) to right premaxilla (RLP)
Width of cleft lip (left)	LL – LPL	Distance from left cleft lip (LL) to left premaxilla (LPL)
Distance between cleft lips	RL – LL	Distance from right cleft lip (RL) to left cleft lip (LL)
Width of the premaxilla	RPL – LPL	Distance from right (RPL) to left (LPL) side of premaxilla
**3D Angle**	**Abbreviation**	**Definition**
Columella deviation angle	CDA	Angle between columella axis and alar base line
Nasal tilt angle	AL – Prn – AL′	Angle between right alare (AL), pronasale (Prn), and left alare (AL′)
Nostril angle (right)	AL – Prn – Sn	Angle between right alare (AL), pronasale (Prn), and subnasale (Sn)
Nostril angle (left)	AL′ – Prn – Sn	Angle between left alare (AL′), pronasale (Prn), and subnasale (Sn)

### Measurement error

Two independent reviewers identified the predefined landmarks manually. Inter-examiner reliability was assessed to determine the consistency of measurements between the two independent examiners. A subset of 47 3D models was measured by both examiners using the same landmark protocol. Intraclass correlation coefficients (ICCs) were calculated using a two-way random-effects model with absolute agreement for single measurements.

### Statistical analysis

Statistical analysis was conducted using IBM SPSS^®^ Statistics software version 28.0 for Windows (SPSS Inc., Chicago, Ill). A linear mixed model was applied to evaluate the influence of therapy (NAM vs. non-NAM) and age on various nasal and perinasal measurements over time. The model included fixed effects for therapy and age, with subject-specific random intercepts to account for individual variability. The percentages of explained variance are also reported. A p-value of < 0.05 was considered statistically significant. In the analysis, no distinction was made between male and female subjects.

Given the retrospective and exploratory design of this longitudinal observational study, no a priori power calculation was performed. The study includes all eligible patients treated within the study period, which may still provide valuable insights despite the lack of a predefined sample size estimation. No formal correction for multiple comparisons was applied, as the analyses were primarily exploratory. To assess inter-examiner reliability, a random subset of 47 models was independently remeasured by two reviewers. Intraclass correlation coefficients (ICCs) were calculated using a two-way random-effects model with absolute agreement for single measurements.

## Results

A total of 72 patients were included in the study, comprising 46 non-syndromic UCLP and 26 non-syndromic BCLP patients. From the initial 82 patients that were recruited, five patients were excluded due to incomplete NAM therapy, four due to relocation during treatment, and one due to parental refusal to allow 3D facial photography.

The 3D models were assessed by two independent reviewers demonstrating excellent inter-rater reliability (ICC = 0.88; 95% CI: 0.82–0.93).

The mean age and standard deviation at the four time points (T_0_–T_3_) are summarized in Table 3. In the BCLP cohort, both groups were imaged at comparable ages at T_0_ and T_2_, although the NAM group had a slightly higher mean age at T_3_ (363.42 ± 98.01 days) compared to the non-NAM group (313.5 ± 34.18 days). At T_1_, no non-NAM patients were available for comparison. In the UCLP cohort, the mean ages were largely comparable between groups at all timepoints, with some variation at T_0_ and T_3_. Notably, the NAM group at T_3_ had a higher mean age (357.35 ± 102.73 days) than the non-NAM group (303.5 ± 24.75 days).

**Table 3:** Mean age and standard deviation (in days) at four timepoints (T0 – T3) for patients with unilateral (UCLP) and bilateral cleft lip and palate (BCLP), stratified by NAM and non-NAM treatment groups (SD = standard deviation)

BCLP		
Timepoint	NAM Therapy (Mean ± SD, days)	Non-NAM Therapy (Mean ± SD, days)
T_0_: 0–50	14.73 ± 20.89	15.5 ± 19.71
T_1_: 51–100	81.71 ± 12.58	—
T_2_: 101–250	138.47 ± 27.85	139.6 ± 29.35
T_3_: 251–650	363.42 ± 98.01	313.5 ± 34.18
**UCLP**		
**Timepoint**	**NAM Therapy (Mean ± SD**,** days)**	**Non-NAM Therapy (Mean ± SD**,** days)**
T_0_: 0–50	13.88 ± 16.19	26.5 ± 33.23
T_1_: 51–100	80.08 ± 16.43	92.67 ± 6.51
T_2_: 101–250	132.48 ± 35.54	128.80 ± 25.14
T_3_: 251–650	357.35 ± 102.73	303.5 ± 24.75

### Unilateral cleft lip and palate (UCLP)

Table 4 presents the median and interquartile range for all 3D distances and 3D angles in both groups across the four defined time points.

**Table 4:** Comparison of 3D Nasal Measurements and Angles Between NAM and non-NAM Groups in Patients with Unilateral Cleft Lip and Palate (UCLP) Across Four Timepoints With Linear Mixed Model Estimates for Therapy and Age Effects (IQR = interquartile range, postOP = postoperative, CDA = columella deviation angle)

**Groups**	**NAM Therapy**				**Non-NAM Therapy**			
**Sample size**	**N = 25**	**N = 25**	**N = 26**	**N = 23**	**N = 2**	**N = 4**	**N = 7**	**N = 4**
**Timepoint (days)**	**T**_**0**_**: 0–50**	**T**_**1**_**: 51–100**	**T**_**2**_**: 101–250**	**T**_3_**: 251–650**	**T**_**0**_**: 0–50**	**T**_**1**_**: 51–100**	**T**_**2**_**: 101–250**	**T**_**3**_**: 251–650**
**3D Distance**	**Median [IQR]**	**Median [IQR]**	**Median [IQR]**	**Median [IQR]**	**Median [IQR]**	**Median [IQR]**	**Median [IQR]**	**Median [IQR]**
AL – AL`	29.22 [3.77]	30.17 [3.36]	29.55 [3.12]	30.41 [3.09]	27.20 [-]	31.06 3.72]	25.78 [3.72]	27.80 [6.02]
Sn – Prn	8.28 [1.24]	9.70 [1.53]	9.55 [1.95]	10.80 [1.66]	7.78 [-]	9.11 [1.30]	9.07 [1.93]	9.71 [3.51]
Sn – Ca	4.26 [1.12]	5.17 [1.23]	5.45 [1.34]	5.76 [1.31]	5.12 [-]	4.72 [1.44]	4.87 [1.99]	5.25 [0.82]
Sn0 – Sn0`	3.53 [1.68]	4.41 [1.02]	4.71 [0.99]	4.53 [1.35]	3.60 [-]	4.24 [0.77]	4.23 [0.48]	4.79 [1.25]
NB – NT	4.54 [1.14]	5.59 [1.23]	5.89 [1.66]	6.51 [1.34]	4.24 [-]	5.22 [1.24]	4.71 [0.55]	6.01 [0.88]
NB` – NT`	3.31 [1.90]	5.66 [2.15]	5.72 [2.10]	5.88 [1.97]	3.13 [-]	5.57 [1.12]	4.64 [0.88]	5.59 [0.35]
AB – Prn	15.41 [1.24]	16.28[1.17]	16.78[2.34]	18.27 [1.26]	13.99 [-]	18.53 [3.09]	16.60 [2.90]	19.54 [3.01]
AB` – Prn	19.78 [2.33]	20.66 [2.86]	20.23 [1.98]	19.85 [3.02]	18.74 [-]	19.64 [3.94]	17.13 [1.53]	17.53 [3.21]
NR – NL	8.16 [1.44]	8.02 [1.08]	8.53 [1.75]	9.28 [1.52]	7.51 [-]	9.62 [3.91]	8.55 [1.15]	9.66 [3.03]
NR` – NL`	15.45 [4.03]	14.75 [4.25]	13.84 [3.71]	11.62 [2.67]	14.64 [-]	15.03 [5.56]	9.92 [2.84]	9.06 [4.40]
LPL – LL	13.07 [7.13]	11.25 [5.66]	10.72 [6.32]	postOP	9.27 [-]	12.38 [5.69]	7.09 [8.29]	postOP
**3D Angle**	**Median [IQR]**	**Median [IQR]**	**Median [IQR]**	**Median [IQR]**	**Median [IQR]**	**Median [IQR]**	**Median [IQR]**	**Median [IQR]**
CDA	48.07 [25.14]	65.31 [15.79]	72.19 [17.16]	85.05 [9.29]	70.56 [-]	69.83 [7.07]	74.01 [12.13]	79.12 [10.44]
AL – Prn – AL`	117.63 [16.04]	106.42 [9.85]	105.90 [12.53]	102.92 [12.51]	113.83 [-]	104.03 [15.98]	95.84 [10.10]	97.78 [17.22]
AL – Prn – Sn	45.37 [13.41]	44.13 [10.34]	46.25 [10.54]	52.00 [8.98]	49.32 [-]	41.26 [22.98]	42.35 [6.77]	43.77 [8.49]
AL`– Prn – Sn	85.63 [24.71]	72.83 [18.42]	69.17 [21.43]	61.61 [8.59]	80.43 [-]	75.82 [30.04]	60.19 [17.68]	55.24 [13.84]
	**Fixed Parameters**				**Ratio**			
	**Therapy**		**Age**					
**3D Distance**	**Estimation**	***p*-value**	**Estimation**	***p*-value**	**%**			
AL – AL`	1.525	0.109	0.013	0.339	65.64			
Sn – Prn	0.507	0.131	0.196	<0.001	33.50			
Sn – Ca	0.226	0.387	0.098	<0.001	23.73			
Sn0 – Sn0`	0.044	0.880	0.023	0.255	12.40			
NB – NT	0.473	0.097	0.176	<0.001	35.99			
NB` – NT`	0.325	0.463	0.125	<0.001	15.20			
AB – Prn	−0.566	0.207	0.262	<0.001	35.41			
AB` – Prn	1.427	0.027	−0.018	0.615	36.80			
NR – NL	−0.640	0.114	0.084	0,001	26.29			
NR` – NL`	1.968	0.029	−0.243	<0.001	57.24			
LPL – LL	0.838	0.579	−0.398	0.045	66.24			
**3D Angle**	**Estimation**	***p*-value**	**Estimation**	***p*-value**	**%**			
CDA	−5.428	0.167	2.202	<0.001	30.28			
AL – Prn – AL`	6.509	0.046	0.408	<0.001	59.12			
AL – Prn – Sn	1.880	0.424	0.407	0.006	28.16			
AL`– Prn – Sn	6.365	0.125	−1,605	<0.001	39.65			

Significant age-related changes were observed across nearly all linear measurements expect for nose width [AL–AL`] (*p* = 0.339), columella thickness [Sn0–Sn0`] (*p* = 0.255), and length of the left nostril [AB´–Prn] (*p* = 0.615). Notably, the length of the left nostril [AB’–Prn] was significantly greater in the NAM group (*p* = 0.027). The cleft-side alar width [NR’–NL’] was also significantly greater in the NAM group (*p* = 0.029).

**Fig. 4:**
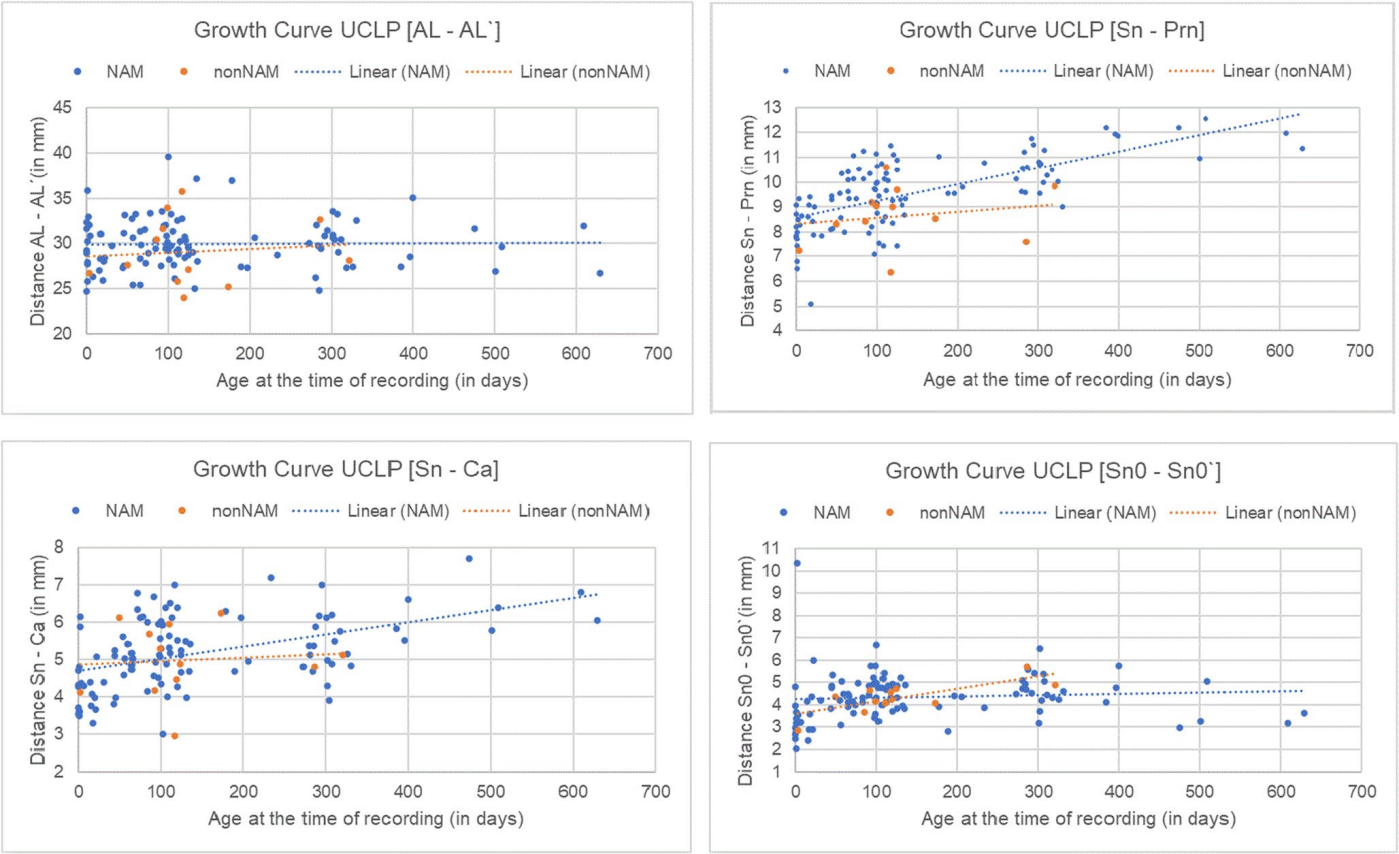
Nasal Development in UCLP. Blue: NAM; orange: non-NAM

In the NAM group, nose protrusion [Sn–Prn] gained length from a median of 8.3 mm at birth to 10.8 mm at two years of age, representing 30% growth (*p* = 0.131). Columella length [Sn–Ca] showed a consistent increase at each time point, with an overall growth of 35% in the investigation period (*p* = 0.387) (Fig. 4).

In the non-NAM group, nose protrusion [Sn–Prn] increased from a median of 7.8 mm at birth to 9.7 mm by the end of the observation period, equivalent to 24% growth (*p* = 0.131). Columella length [Sn–Ca] had overall growth of 4%. At birth, the non-cleft right nostril height [NB–NT] had a median of 4.2 mm, higher than the cleft-side left nostril height [NB’–NT’] with a median of 3.1 mm. Both increased over time, with the right nostril height reaching 6.0 mm and the left nostril height reaching 5.6 mm. The right nostril length [AB–Prn] on the non-cleft side was significantly smaller at birth, with a median of 14.0 mm, compared to the left cleft-side nostril length [AB’–Prn] of 18.7 mm. Over time, the non-cleft-side nostril length increased to a median of 19.5 mm, while the cleft-side nostril length decreased to 17.5 mm, ultimately becoming smaller than the right nostril.

**Fig. 5:**
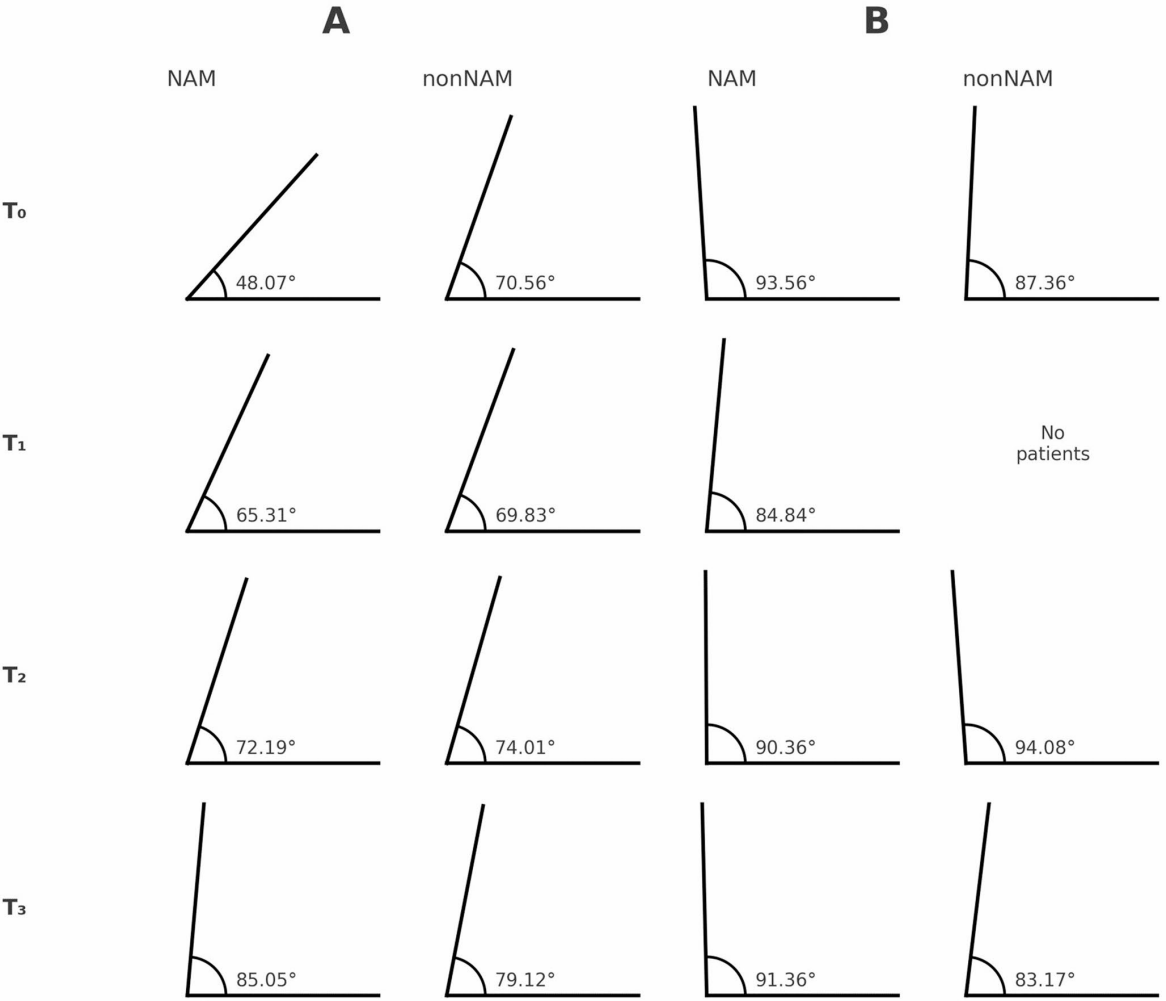
Graphical Representation of the Change in Columella Deviation Angle Over Time (T_0_–T_3_) in Left-sided UCLP (A) and BCLP (B) for the NAM and non-NAM Groups

The columella deviation angle increased with age (*p* < 0.001). The NAM group the columella deviation angle showed an increase over time, with median values progressing from 48.1° at birth to finally 85.1°, representing 76% growth (*p* = 0.167) (Fig. 5). In the non-NAM group, the columella deviation angle showed an increase, from a median of 70.6° at birth to 79.1° at the final observation point, representing 12% growth (*p* = 0.167). Other angular measures, such as the angle of the right [AL–Prn–Sn] and left [AL’–Prn–Sn] nostril, also improved with age (*p* = 0.006; *p* < 0.001), though the differences between therapy groups were not statistically significant (*p* = 0.424, *p* = 0.125).

Variance analysis from the linear mixed model indicated that subject-level differences accounted for a substantial proportion of total variability in some parameters. For instance, nose width [AL–AL’] had a variance component of 65.64%, highlighting individual anatomical variability. Lower variance was observed in parameters like columella thickness [Sn0–Sn0’] (12.40%), suggesting more uniform responses across patients.

### Bilateral cleft lip and palate (BCLP)

Table 5 provides the median and interquartile range for all 3D distances and 3D angles across the four time points for both study groups.

**Table 5:** Comparison of 3D nasal measurements and angles between NAM and non-NAM groups in patients with bilateral cleft lip and palate (BCLP) across four timepoints with linear mixed model estimates for therapy and age effects (IQR = interquartile range, postOP = postoperative, CDA = columella deviation angle)

Groups	NAM Therapy				Non-NAM Therapy			
Sample size	*N* = 15	*N* = 14	*N* = 15	*N* = 12	*N* = 4	*N* = 0	*N* = 5	*N* = 6
Timepoint (days)	T_0_: 0–50	T_1_: 51–100	T_2_: 101–250	T_3_: 251–650	T_0_: 0–50	T_1_: 51–100	T_2_: 101–250	T_3_: 251–650
**3D Distance**	**Median [IQR]**	**Median [IQR]**	**Median [IQR]**	**Median [IQR]**	**Median [IQR]**	**Median [IQR]**	**Median [IQR]**	**Median [IQR]**
AL – AL`	30.63 [3.72]	33.46 [5.97]	32.74 [4.41]	34.27 [6.05]	28.92 [1.89]	-	30.89 [3.97]	34.05 [5.13]
Sn – Prn	6.22 [1.98]	7.44 [1.69]	7.89 [2.26]	10.94 [1.66]	6.98 [1.86]	-	7.56 [2.17]	9.93 [2.93]
Sn – Ca	2.11 [1.58]	3.48 [1.39]	3.58 [1.42]	5.15 [1.81]	2.38 [2.04]	-	3.51 [1.52]	4.10 [1.59]
Sn0 – Sn0`	5.62 [2.23]	5.01 [1.38]	5.05 [0.88]	5.60 [1.92]	5.28 [2.26]	-	4.59 [1.62]	5.73 [1.46]
NB – NT	3.44 [1.21]	5.17 [2.48]	5.22 [2.15]	5.35 [1.17]	4.73 [3.31]	-	4.52 [1.30]	4.87 [0.74]
NB` – NT`	3.95 [2.85]	5.26 [2.05]	5.93 [2.01]	5.79 [1.26]	3.94 [2.03]	-	4.75 [1.13]	4.97 [0.74]
AB – Prn	17.61 [2.38]	22.20 [3.38]	21.77 [4.89]	19.76 [2.43]	18.35 [3.48]	-	19.01 [3.12]	19.60 [3.54]
AB` – Prn	19.05 [4.50]	20.54 [4.99]	18.10 [6.33]	19.83 [3.17]	17.70 [5.75]	-	19.13 [5.76]	19.46 [1.23]
NR – NL	10.55 [3.13]	12.99 [4.45]	13.16 [4.41]	11.32 [2.76]	10.52 [3.72]	-	10.99 [3.71]	11.66 [3.75]
NR` – NL`	10.85 [4.60]	11.90 [5.69]	10.88 [5.73]	11.98 [1.86]	11.33 [3.28]	-	10.87 [5.76]	13.12 [2.80]
RL – RPL	9.79 [9.10]	9.81 [6.26]	11.57 [2.95]	postOP	5.48 [4.65]	-	7.40 [6.34]	postOP
LPL – LL	9.25 [7.96]	9.01 [5.66]	8.98 [6.41]	postOP	7.86 [7.35]	-	7.42 [7.58]	postOP
RL – LL	23.13 [4.17]	23.66 [6.10]	21.57 [5.70]	postOP	21.91 [4.82]	-	23.86 [4.65]	postOP
RPL – LPL	12.21 [4.21]	12.56 [3.17]	13.53 [1.74]	postOP	11.98 [5.71]	-	12.05 [3.41]	postOP
**3D Angle**	**Median [IQR]**	**Median [IQR]**	**Median [IQR]**	**Median [IQR]**	**Median [IQR]**	**Median [IQR]**	**Median [IQR]**	**Median [IQR]**
CDA	93.56 [31.05]	84.84 [18.91]	90.36 [10.16]	91.39 [8.52]	87.26 [11.55]	-	94.08 [30.08]	83.17 [11.05]
AL – Prn – AL`	119.11 [16.60]	110.43 [16.89]	108.38 [11.83]	114.83 [12.66]	103.76 [15.64]	-	106.74 [10.55]	111.68 [9.30]
AL – Prn – Sn	69.90 [15.78]	61.01 [10.72]	66.27 [12.02]	64.44 [8.26]	57.69 [20.44]	-	50.24 [7.00]	60.49 [6.61]
AL`– Prn – Sn	72.31 [16.32]	71.68 [9.92]	66.73 [5.78]	66.59 [4.72]	63.55 [18.95]	-	62.21 [13.65]	68.67 [11.33]
	**Fixed Parameters**				**Ratio**			
	**Therapy**		**Age**					
**3D Distance**	**Estimation**	***p*-value**	**Estimation**	***p*-value**	**%**			
AL – AL`	1.204	0.337	0.112	0.137	38.10			
Sn – Prn	0.069	0.146	0.338	< 0.001	20.06			
Sn – Ca	0.440	0.177	0.181	< 0.001	27.45			
Sn0 – Sn0`	0.374	0.385	0.085	0.011	17.36			
NB – NT	0.248	0.599	0.097	0.010	14.66			
NB` – NT`	0.792	0.074	0.103	0.001	25.57			
AB – Prn	0.700	0.443	0.080	0.189	29.14			
AB` – Prn	1.022	0.298	−0.011	0.845	44.01			
NR – NL	0.389	0.633	0.026	0.653	23.34			
NR` – NL`	−0.437	0.684	−0.002	0.971	40.10			
RL – RPL	2.021	0.251	0.139	0.524	65.01			
LPL – LL	0.752	0.697	−0.194	0.318	78.04			
RL – LL	2.214	0.211	−0.342	0.265	28.27			
RPL – LPL	1.395	0.196	0.038	0.739	74.77			
**3D Angle**	**Estimation**	***p*-value**	**Estimation**	***p*-value**	**%**			
CDA	4.789	0.300	0.177	0.618	14.69			
AL – Prn – AL`	6.467	0.170	−0.059	0.811	47.63			
AL – Prn – Sn	8.883	0.014	−0.252	0.269	28.49			
AL`– Prn – Sn	1.987	0.778	−0.298	0.143	14.28			

Significant age-related increases were observed in several nasal dimensions, including nose tip protrusion [Sn–Prn] (*p* < 0.001), columella length [Sn–Ca] (*p* < 0.001), columella thickness [Sn0–Sn0’] (*p* = 0.011), and the heights of the right [NB–NT] and left [NB’–NT’] nostrils (*p* = 0.010, *p* = 0.001).

**Fig. 6:**
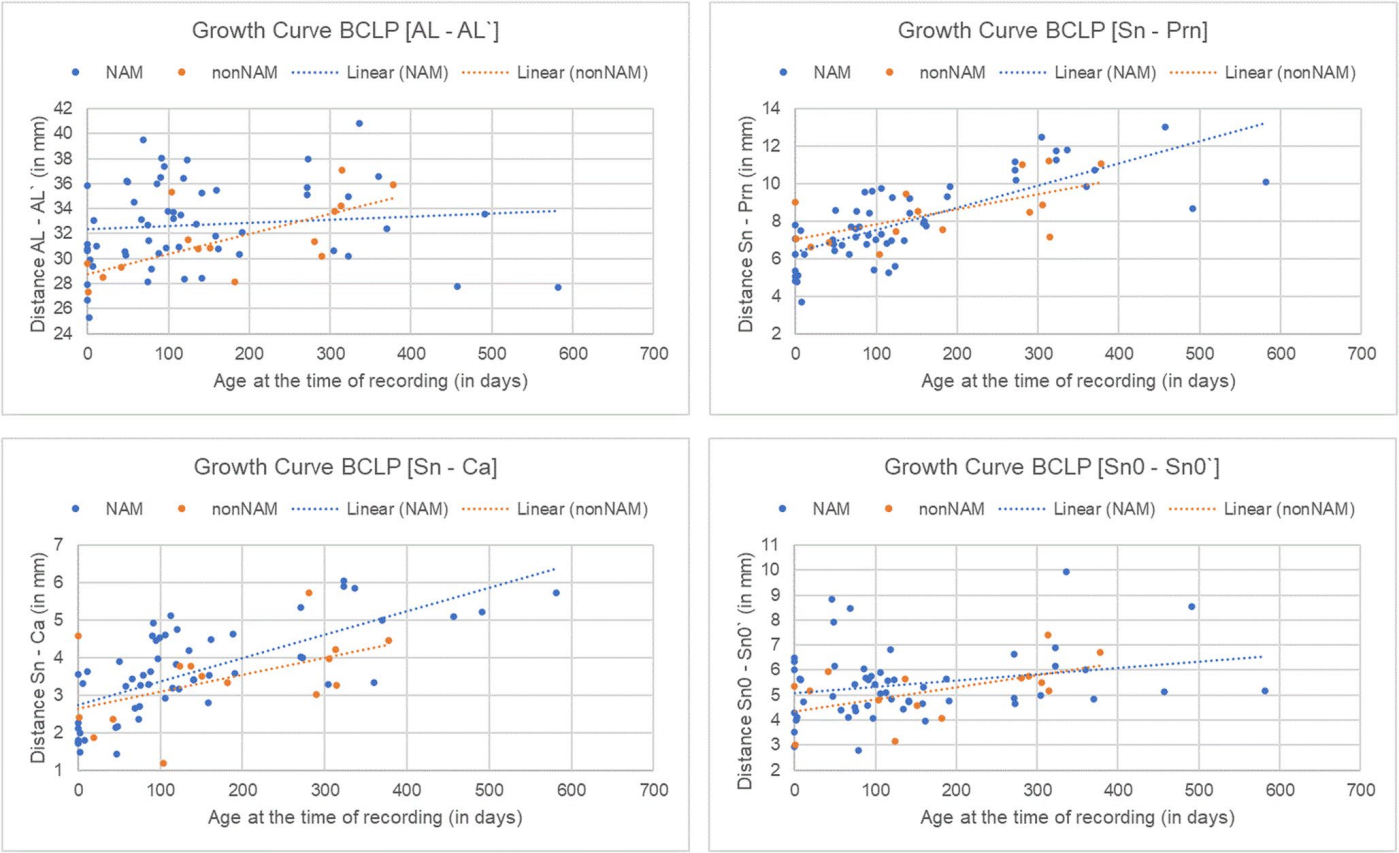
Nasal Development in BCLP. Blue: NAM; orange: non-NAM

In the NAM group, nasal protrusion [Sn–Prn] showed a consistent increase from a median of 6.2 mm to finally 10.9 mm (+ 4.7 mm, + 42%, *p* = 0.146) (Fig. 6). The columella length [Sn–Ca] also showed an increase from a median of 2.1 mm to 5.2 mm (+ 3.1 mm, *p* = 0.177). The right nostril length [AB–Prn] initially increased from a median of 17.6 mm to 19.8 mm (+ 13%, *p* = 0.443). The left nostril length [AB’–Prn] showed a small increase from a median of 19.1 mm to 19.8 mm (4%, *p* = 0.298). The right [NR–NL] increased from 10.6 mm to 11.3 mm (+ 7%, *p* = 0.633) and the left [NR′–NL′] from 10.8 mm to 12.0 mm (+ 11%, *p* = 0.684). The width of the right cleft lip [RL–RPL] increased from 9.8 mm to 11.6 mm (*p* = 0.251), while the width of the left cleft lip [LPL–LL] slightly decreased from 9.3 mm to 9.0 mm (*p* = 0.697). These measurements showed wide interquartile ranges, indicating substantial anatomical variability within the cohort.

In the non-NAM group, similar trends were observed. Nasal protrusion [Sn–Prn] increased from 7.0 mm to 9.5 mm (+ 2.5 mm, *p* = 0.146), and columella length [Sn–Ca] grew from 2.4 mm to 4.1 mm (+ 1.7 mm, *p* = 0.177).

The columella deviation angle (CDA) in the NAM group decreased from a median of 93.6° to 91.4° (Fig. 4). In terms of 3D angles, the CDA in the non-NAM group decreased from a median of 87.3° to 83.2° (*p* = 0.300).

The width of the left cleft lip [AL–AL’] had a variance component of 78.0%, and the width of the premaxilla [RPL–LPL] had a variance component of 74.8%, highlighting considerable individual anatomical variability. In contrast, lower variance was observed in parameters such as columella thickness [Sn0–Sn0’] (17.36%), suggesting more uniform responses across patients.

## Discussion

This study presents our clinical outcomes for children with UCLP and BCLP who were treated following a standardized protocol, concentrating on the age from 0 to 2 years. This protocol included either non-NAM or NAM therapy using Grayson’s technique in the presurgical phase, as previously described [6, 24].

### 3D imaging and landmark methodology

Imaging plays an important role in therapy planning, progress documentation, and effectiveness monitoring in patients with CLP. Traditionally, 2D photography has been the most widely used tool due to its simplicity and low cost [11, 12]. However, it is limited by factors like lighting, head position, and camera distance, leading to potential measurement errors. To overcome these limitations, 3D surface imaging has gained prominence. 3D photography, particularly stereophotogrammetry (e.g., 3dMD), offers high-resolution, non-invasive imaging with minimal motion artifacts. Computer tomography and magnetic resonance imaging, though useful, are generally unsuitable for growth monitoring due to radiation exposure and long scan times.

Despite the increasing application of 3D scanning in clinical practice, a lack of standardized 3D facial reference data, especially for CLP patients, remains a barrier. To address this, future research should aim to build accessible image databases using 3D photographs to establish reliable datasets.

The landmarks used in this study have been widely used in previous studies on three-dimensional facial analysis and are therefore well suited for comparative purposes [23, 28]. The distances and angles derived from them have already been examined for reproducibility in earlier studies by Ritschl et al. and Swennen [26, 27]. However, no angles in the sagittal plane were defined or studied. To describe nasal growth even more precisely using angles, it would be beneficial in the future to include sagittal plane angles as well, as this would provide additional spatial information in three-dimensional analysis. In this study, the nasion was not defined as a landmark, which made measurements in the sagittal plane impossible.

The measurement of 3D photographs using stereophotogrammetry and laser scanners has been shown to be as accurate as direct anthropometry [29–31]. An issue in 3D facial morphology analysis is the lack of universally accepted reference planes. Many studies have attempted to define suitable planes for precise anthropometric measurements. For example, Mishima et al. defined the x-y plane using the nasion and the left and right endocanthion [32]. Bacher et al. proposed a plane defined by the tragus and the midpoint of the intercanthal distance (en-en) [33, 34]. To date, however, no standard reference plane has been established. As a result, many researchers choose not to provide x/y/z coordinates, instead reporting results as two-dimensional distances and angles. Unfortunately, this leads to a loss of valuable spatial information [35]. Moreover, this limits the comparability between studies.

Artificial intelligence is also emerging as a valuable tool in craniofacial analysis. A study performed by Berends et al. shows an automated system that can perform landmark positioning in the face achieving comparable accuracy and precision as trained examiners [36]. In the future, integrating such systems may help perform landmark-based analysis quickly and efficiently, offering examiners a user-friendly and time-saving solution, as well as increasing reproducibility.

### Impact of NAM therapy

In the case of bilateral clefts, a shortened columella often leads to a flattening of the tip of the nose. The focus of NAM therapy in UCLP is therefore mainly on straightening the columella and in the case of BCLP on elongating the columella [6, 9, 37]. Liou et al. showed that NAM significantly improved both vertical and horizontal nasal asymmetry before primary cheiloplasty, although the nose remained asymmetrical afterward due to the unrepaired cleft lip and nose [38]. Similarly, our study found that NAM therapy increased nostril height and significantly reduced nostril width (*p* = 0.029) and nostril length (*p* = 0.027) on the cleft side, while increasing nostril length on the non-cleft side. These changes resulted in improved nasal symmetry on the affected cleft side of the nose, and a reduced deviation in the cases of ULCP. Our null hypothesis, that NAM has no significant influence on nasal morphology, has therefore been refuted. However, even after two years, the cleft-side nostril remained smaller in height, wider in width, and longer in length compared to the non-cleft side. The primary goal of NAM was to mold the nasal cartilage and narrow the alveolar cleft, facilitating subsequent surgical repair, rather than achieving complete symmetry. Regarding focusing on columella straightening in UCLP patients, Gomez et al. showed a significant reduction of cleft columella deviation with an increase in columella length [39]. Our study came to similar findings. Growth curves indicated more pronounced lengthening of the columella and greater nasal tip protrusion in the NAM group, favoring symmetry and aesthetics compared to the non-NAM group.

Nasal tip protrusion measurements performed at one year of age in a healthy cohort by Farkas et al. were reported as 10.1 mm in male patients and 10.2 mm in female patients. Among nasal measurements (including nose width, nose height, nasal bridge length, nasal tip protrusion, and ala length), nasal tip protrusion was proportionally the least developed at this age. This was followed by marked early growth—between ages 1 and 3 years in females and 1 and 4 years in males—resulting in a 46% increase in both sexes [40]. While a direct comparison between groups is not entirely possible due to differences in time periods, there appears to be less growth in the UCLP group despite the patients undergoing NAM therapy. Additionally, the CDA increased by 76% in the NAM group, reaching a median of 85.0° after two years, which is a non-significant trend toward symmetry In contrast, the non-NAM group saw a 12% increase in the CDA, reaching a median of 79.1° after the same period. Furthermore, in the NAM group, the cleft-side nostril angle decreased while the non-cleft-side nostril angle increased, resulting in a more symmetrical nasal shape. In contrast, the non-NAM group demonstrated greater asymmetry, with more pronounced differences in nostril angles between the two sides.

Rau et al. reported a statistically significant increase in the height of both right and left nostrils in bilateral cleft patients treated with NAM therapy [6]. Similarly, our study found that NAM patients had greater increases in nostril height compared to the non-NAM group (left:

*p* = 0.074, right: *p* = 0.60). By the end of the observation period, the right and left nostril lengths (right: *p* = 0.44; left: *p* = 0.30) and widths (right: *p* = 0.633; left: *p* = 0.684) were nearly identical in both therapy groups, showing a tendency toward improved symmetry, although statistical significance was not reached. The results of this study revealed that the nasoalveolar molding improved the nasal asymmetry in cleft patients. The tendencies were observed in all dimensions—in nostril height, nostril length, and nostril width.

In the NAM cohort, the impact of columella straightening is illustrated in Fig. 3 (*p* = 0.300). In contrast, the non-NAM group showed a decrease in CDA throughout the observation period, resulting in poorer symmetry. In an earlier investigation our research group compared nasal growth in NAM-treated BCLP patients with a healthy control group, demonstrating that NAM therapy significantly lengthens the columella. However, even with the effective NAM treatment for bilateral clefts, patients will not achieve the nasal dimensions typical of healthy individuals [23]. Future studies could consider adding a healthy patient cohort as a third group alongside the NAM and non-NAM groups. Nevertheless, it should also be noted that a statistically significant increase in the angle of the right nostril was shown in the NAM group (*p* = 0.014).

The primary advantages of NAM over conventional palatal plate therapy for unilateral clefts are enhanced aesthetics and improved symmetry relative to the unaffected side. By gradually reshaping and aligning the nasal and alveolar structures, NAM improves nasal tip projection, lengthens the columella, and decreases the visual disparity between the cleft and non-cleft sides [6, 9, 37]. Namdar et al.’s review highlighted several nasal growth benefits associated with NAM therapy, including the lengthening of the columella, repositioning of deformed nasal cartilage, and enhanced nasal symmetry [41].

This observational period reflects short-term outcomes rather than long-term results; however, both our findings and those of others demonstrate beneficial initial results. For instance, Mancini et al. conducted a 3D analysis of 20 consecutive cases with UCLP, reporting significant improvements in nasal projection, width, symmetry, and columella length [9]. Van der Heyden et al. reviewed the long-term effects of NAM on nasal symmetry in unilateral clefts and found heterogeneous results. One study showed significant improvements with NAM plus surgery compared to surgery alone, another reported no benefit, and six further studies produced mixed outcomes without consistent effects [42]. Barillas et al. reported that significant improvements in nasal symmetry after NAM persisted at least until the age of nine years [7] while AlHayyan et al. found no significant long-term differences in midface symmetry between NAM-treated UCLP patients and those without presurgical orthopedics [43].

Other studies further indicated relapse of initially favorable outcomes [38, 44, 45]. Relapse following surgical correction in patients with CLP remains a significant challenge despite advancements in surgical techniques and postoperative management. One of the primary challenges in treating CLP patients is adjusting the ongoing growth and development of the facial skeleton and soft tissues. As children grow, originally favorable surgical outcomes may be changed by growth variations, particularly in the midface. Restricted maxillary growth, which is common in CLP patients, often contributes to the recurrence of both functional and aesthetic issues, such as malocclusions or nasal deformities [17, 46, 47].

An additional challenge is differentiating whether deformities arise from an intrinsic tissue deficiency or as secondary effects of surgery. CLP repair involves the precise closure of multiple tissue layers, but the healing process can lead to scar tissue formation, which lacks the elasticity and growth capacity of normal tissue. Scar contracture can create tension, misaligning the repaired structures and affecting their function, which contributes to relapse [48]. The timing and technique of surgical interventions are very important to achieving stable outcomes. Early surgical intervention can address aesthetic concerns rapidly but may interfere with natural growth processes, potentially necessitating future surgeries. In contrast, delayed surgeries may permit more refined tissue handling but risk missing critical windows for optimal functional development [49]. Therefore, carrying out long-term studies on the NAM technique is essential. A recent study of CLP patients shows that timing remains an important variable throughout therapy since even metabolic skeletal development could be influenced [50].

Further challenges in presurgical orthofacial treatment include the development of “mega-nostrils,” notably pronounced in unilateral clefts, where the cleft-side nostril becomes noticeably enlarged relative to the unaffected side. This may arise from excessive nasal cartilage pressure or mispositioning of the stent. Some centers aim for intentional overcorrection to offset postoperative growth relapse, though this approach remains debated due to limited supporting data [51]. We did not observe the development of “mega-nostrils” following the molding procedure.

Additionally, securing the NAM device with adhesive strips may lead to facial dermatitis in infants, and incorrect placement of the nasal bridge or palatal plate can result in pressure points and ulcerations within the oral and nasal cavities. This highlights the need for careful patient monitoring [51]. With our team providing supervision once a week, parents assumed responsibility for daily taping, underscoring the critical role of parental support and adherence in the success of NAM. Although NAM therapy may impose significant stress on caregivers, who bear considerable responsibility, Roth et al. report that all surveyed parents felt they were positively contributing to their child’s care through NAM therapy. Overall, parents said they felt well-supported and informed throughout the process [28]. In summary, while NAM therapy is both time- and cost-intensive, its benefits for patients and caregivers are substantial and outweigh the associated burdens [28, 52, 53]. Nonetheless, a long-term study investigating band-aid intolerance in patients undergoing NAM therapy could improve the overall quality of NAM therapy, and help develop strategies to minimize discomfort and optimize treatment outcomes in cleft lip and palate patients.

### Methodological considerations

In longitudinal growth studies, age consistency across comparison groups is important to ensure valid interpretation of developmental differences. While the current study defined standardized timepoints (T_0_–T_3_) by age ranges, variability in the actual mean ages at which 3D images were captured was observed, particularly at later stages (T_3_). Moreover, the relatively large standard deviations in age, especially in later timepoints (T_3_), indicate a wide spread in when patients returned for imaging. This heterogeneity likely reflects real-world variability in clinical follow-up and can impact the interpretation of longitudinal growth patterns. Although we accounted for chronological age as a fixed covariate in the linear mixed model, such broad age ranges may still mask subtle developmental differences between patients and reduce the sensitivity to detect treatment-specific effects. For example, facial structures like the columella, nasal base, and alar width undergo rapid changes in infancy, and a 2–3-month difference at this age can be physiologically meaningful.

Moreover, in the statistical analysis, therapy and age were included as fixed effects. However, the potential interaction between therapy and age was not explored. Investigating this interaction could provide deeper insights into whether the effect of NAM therapy varies across different stages of early development. Because multiple outcomes were analyzed without formal correction, the risk of type I error cannot be excluded, and the findings should be considered hypothesis-generating. Future studies with larger samples and longer follow-up should incorporate interaction terms and apply adjustments for multiple comparisons. There were noticeable variations in the mean ages at which 3D images were captured between groups, introducing potential confounding factors when comparing morphological outcomes. Later imaging may coincide with more advanced growth, potentially overestimating treatment effects. This underlines the importance of standardized timing in longitudinal imaging, especially in growth-sensitive regions like the nasal complex. Future studies may benefit from narrower age windows or stratified sampling to ensure closer temporal alignment.

Participants were not stratified by gender, as White et al. found no sex-specific differences in nasal or oral regions or in facial angles in three-month-old infants. However, they reported that larger facial dimensions, such as facial height and nasal width, correlated significantly with body size. Including weight, height, and weight-for-length z-scores at imaging could therefore improve future data comparability [54].

Ethnicity is another factor influencing facial morphology. Agbolade et al. demonstrated ethnic effects on facial features using 3D stereophotogrammetry [55]. In our study, all eligible participants were included regardless of ethnicity, but future research should consider stratification to enhance comparability and interpretability.

### Limitations

One limitation of this study was the small sample size of the non-NAM group, raising concerns about its representativeness. The limited size resulted from the high acceptance of NAM therapy among cleft patients at the Klinikum rechts der Isar, leaving few patients untreated with NAM. Due to the rather small cohort, statistical significance oftentimes cannot be achieved. However, the absence of statistical significance in our case does not mean there is no clinical relevance. For a larger cohort, collaboration with other cleft centers may be favorable. Then, however, different treatment protocols and surgeons can be a problem.

## Conclusion

This study highlights the short-term effectiveness of NAM therapy in improving nasal morphology and symmetry in infants with UCLP and BCLP. Specifically, NAM-treated patients demonstrated greater increases in columella length, nasal tip protrusion, and more balanced nostril dimensions, particularly in the cleft-side nostril. These morphological changes may offer clinical benefits, such as facilitating improved surgical outcomes during primary cheiloplasty and enhancing long-term nasal aesthetics and stability.

## Data Availability

Data is provided within the manuscript. If further information is needed, please contact the corresponding author.
